# Redundant Configuration Method of MEMS Sensors for Bottom Hole Assembly Attitude Measurement

**DOI:** 10.3390/mi15060804

**Published:** 2024-06-19

**Authors:** Yu Zheng, Lu Wang, Fan Zhang, Zulei Yang, Yuanbiao Hu

**Affiliations:** Faculty of Engineering Technology, China University of Geosciences (Beijing), Beijing 100083, China; 2002220053@email.cugb.edu.cn (Y.Z.); zf19992023@163.com (F.Z.); 2002220054@email.cugb.edu.cn (Z.Y.); hyb@cugb.edu.cn (Y.H.)

**Keywords:** MEMS redundancy, Kalman filtering, data fusion, measurement while drilling

## Abstract

Micro-electro-mechanical systems inertial measurement units (MEMS-IMUs) are increasingly being employed for measuring the attitude of bottom hole assemblies (BHAs). However, the reliability and measurement precision of a single MEMS-IMU may not meet drilling’s stringent needs. Redundant MEMS-IMU systems can effectively enhance the reliability and precision. This paper proposes a redundant configuration method for MEMS sensors tailored to BHA attitude measurement. Firstly, based on reliability theory and a cost-benefit analysis, considering factors such as cost, size, and reliability, the optimal number of sensors in the redundant system was determined to be six. Considering the structural characteristics of the BHA, a hollow hexagonal prism-shaped redundant configuration scheme was proposed, ensuring the circulation of drilling fluid within the drill pipe. Next, by employing Kalman filtering to integrate the output data from the six sensors, a virtual IMU (VIMU) was formed. Finally, experimental verification was carried out. The results confirmed that, after redundancy implementation, the velocity random walk of the accelerometer decreased by an average of 58% compared to a single MEMS-IMU, and bias instability was reduced by an average of 54%. The angular random walk of the gyroscope decreased by an average of 58%, and bias instability was reduced by an average of 37%. This research provides a theoretical foundation for enhancing the precision and reliability of BHA attitude measurements.

## 1. Introduction

In the realm of rotary steerable drilling, attitude measurement technology stands as one of the core competencies, empowering operators to promptly ascertain the exact location and orientation of the bottom hole assembly (BHA). By continuously gathering spatial position information and attitude data of the BHA, the drilling process can be meticulously monitored. Inertial measurement units (IMUs) serve the purpose of measuring attitude. MEMS sensors, with their attributes of miniaturization, high sensitivity, and low power consumption, have been widely adopted in various precision measurement applications. They not only play a vital role in fields such as moisture detection [1] but also exhibit substantial potential in the advancement of soft robotics technology [2]. Micro-electro-mechanical systems inertial measurement units (MEMS-IMUs) offer advantages over fiber-optic gyros, such as lower costs and smaller sizes. Nonetheless, in the intricate and challenging subterranean working environment, the reliability and measurement accuracy of individual MEMS sensors often fall short of operational requirements. Implementing a redundant system composed of multiple MEMS sensors effectively addresses these shortcomings by bolstering both reliability and measurement precision [3,4,5,6,7,8].

The reliability of a redundant system is chiefly determined by the sensor configuration scheme and the number of sensors, areas that have garnered significant attention from scholars. Reference [9] compares the reliability of various redundant configuration schemes, concluding that, with a maximum of six sensors, the order of configurations from highest to lowest reliability is as follows: six-sensor conical, five-sensor conical, six-sensor orthogonal, four-sensor conical, and five-sensor orthogonal. Reference [10] selects nine sensors as the basis, and a novel orthogonal rotation configuration is proposed, which distributes two sensors along each of the three orthogonal axes and adds a diagonal axis with three sensors. This configuration is compared with an alternative eighteen-faced polyhedron arrangement using nine sensors, demonstrating higher reliability for the orthogonal rotating configuration. Reference [11] introduces a new tri-rotational strapdown inertial navigation system designed for the automotive navigation sector. This system allows for independent rotation along all three axes, with each axis equipped with two accelerometers and two gyroscopes, significantly boosting overall system reliability. These studies exemplify the ongoing advancements in optimizing sensor arrangements for enhanced performance in various applications.

Redundant configuration strategies also have an impact on the measurement accuracy [12,13,14,15,16,17]. Reference [12] designed and analyzed two different conical configuration redundant configurations of 4, 5, 6, and 8 gyro non-orthogonal arrays, revealing that the cone mounting angle has a substantial effect on the navigation performance of MEMS-IMUs. Reference [13] designed redundant systems with 4, 5, and 6 gyroscopes and proposed an optimized Kalman filter fusion algorithm, which enhanced the system’s accuracy. An evaluation criterion for the redundant gyroscope system was established to assess the performance of the algorithm. Regarding data fusion algorithms, Reference [18] introduced a method that combines multiple uncorrelated gyroscopes to improve the precision of an MEMS gyroscope array. By employing a Kalman filter to fuse the output signals from multiple unrelated sensors, the study demonstrated, through experiments, that this approach can effectively boost the accuracy of MEMS gyroscopes.

Indeed, much of the existing research on MEMS sensor redundancy has focused on sectors such as aerospace, marine navigation, and automotive guidance, and when applying these designs to drilling operations, there are many specific engineering application problems. Firstly, drilling tools operate within confined spaces, which puts a constraint on the volume of redundant MEMS-IMUs. The increase in the number of sensors also brings an increase in cost and power consumption. In fact, as the number of sensors increases, system reliability growth is not linear. Beyond a certain point, further increases in sensor count yield marginal gains in reliability. Consequently, in the context of drilling, there is a crucial need to strike a delicate balance between enhancing reliability and managing costs and physical parameters such as size. This underscores the necessity for tailored designs that cater specifically to the unique demands and constraints inherent to the drilling environment.

Furthermore, while configurations based on regular polyhedrons or conical shapes have their merits, they pose two significant challenges in the context of drilling operations. Firstly, drill strings typically function as conduits for drilling fluid circulation, necessitating an internal hollow space. As mentioned above, redundant configurations, with their solid or conically stacked designs, do not accommodate this requirement for an empty central bore within the drill string. Secondly, the above two redundant configuration schemes belong to the inclined non-orthogonal configuration, which not only complicates manufacturing and installation processes but also introduces greater susceptibility to errors. Due to limitations in installation techniques, the actual installed angles will inevitably deviate from the theoretically designed angles, and such deviations are notably larger in non-orthogonal structures compared to orthogonal ones. These installation inaccuracies can severely degrade the precision of attitude measurements.

Given the preceding discussion, this paper proposes a tailored MEMS sensor redundancy configuration scheme for drilling tools, accompanied by the design of a corresponding redundant mechanical structure and an exploration of data fusion algorithms, to enhance reliability through strategic redundancy configurations and to improve accuracy via data fusion methodologies. In Section 2, the optimal number of redundant sensors is obtained, and the redundant configuration scheme of a hollow hexagonal prism is designed. In Section 3, a Kalman filtering approach is designed and implemented for the fusion of sensor data. In Section 4, experimental verification is carried out. The last part is the discussion and conclusion.

## 2. Design of Redundant Configuration Scheme for MEMS Sensors

### 2.1. Research on the Number of Redundant Sensors

In reliability theory, reliability is defined as the probability that a system will function properly under specified conditions and within the expected duration. The x represents the number of times the inertial sensor has failed. It is commonly assumed that x is a random variable, and the number of failures x that occur within time t follows a Poisson distribution, which can be represented by the following equation [9]:

(1) $$h\left(x\right)=\frac{{\left(\lambda t\right)}^{x}\cdot e^{-\lambda t}}{x!},$$

where λ is the failure rate, λt representing the average number of failures within time t. When x is equal to 0, $p(0)=e^{-\lambda t}$; it represents the probability of no failure occurring in time, denoted as follows:

(2) $$R\left(t\right)=e^{-\lambda t},$$

In engineering, the reliability of a sensor is expressed as the mean time between failures (MTBF):

(3) $$T_{MTBF}=\int_{0}^{\infty }R(t)dt,$$

It is assumed that each sensor in the redundant system operates independently, such that a failure in one sensor does not affect the others; the reliability of the non-redundant system and the mean time between failures (MTBF) are respectively defined as follows:

(4) $$R_{1}=R\left(t\right)=e^{-\lambda t},$$

(5) $$T_{1}=\int_{0}^{\infty }e^{-\lambda t}dt=\frac{1}{\lambda },$$

The redundant configuration scheme designed in this paper uses three-axis sensors, and each sensor can work independently, so the minimum number of sensors that the redundant system can operate is 1. In a multi-sensor redundant system, assuming that the number of inertial sensors is n and the reliability of all sensors is $R_{1}$, the probability of failure of one sensor is $1-R_{1}$. The reliability and mean time between failures of the redundant system composed of n sensors can be expressed as follows:

(6) $$R_{n}=1-{\left(1-R_{1}\right)}^{n},$$

(7) $$T_{n}=\int_{0}^{\infty }R_{n}dt,$$

Define redundancy system reliability as θ:

(8) $$\theta =\frac{T_{n}}{T_{1}},$$

Although the reliability of a redundant system tends to increase as the number of sensors is added, this concurrently leads to increments in the overall weight, volume, and cost of the system. Consequently, more sensors do not necessarily equate to better performance. Beyond a certain threshold, the incremental increase in reliability from adding more redundancies becomes progressively smaller, whereas costs rise rapidly, exemplifying the economic phenomenon of “diminishing marginal utility”. Hence, in devising a redundant configuration scheme, the focus should not be exclusively on maximizing system reliability but must also account for factors such as system cost, volume, and others. The cost constraint can be introduced to establish the performance constraint index P:

(9) $$P=\frac{\theta }{f(n)}=\frac{\int_{0}^{\infty }\left[1-{\left(1-e^{-\lambda t}\right)}^{n}\right]dt}{f(n)\int_{0}^{\infty }e^{-\lambda t}dt},$$

where n represents the number of sensors, f(n) is a cost function, and f(n) represents the increase multiple of the cost of a redundant system compared with a non-redundant system as the number of sensors n changes. The meaning of P is that, considering the two factors of reliability improvement and cost improvement brought by the increase in the number of sensors, it is positively proportional to the reliability of the redundant system and inversely proportional to the cost. Its practical engineering significance is that it is hoped that the redundant system can achieve relatively high reliability improvement at the lowest possible cost. At the same time, considering the system volume factor in combination with the narrow environmental conditions when drilling tools work, this paper only considers the calculation of the P index value in the interval range of n∈[1, 10]. When the P value is the largest, n is the optimal choice for the number of sensors.

The functional relationship between n and f(n) is usually not linear. When a non-redundant system changes to a redundant system, the increased costs include the processing costs of the redundant system structure and the costs of sensors, circuit boards, etc. Therefore, when the system changes from a non-redundant state to a redundant state, the cost rises rapidly. Later, as the number of redundant sensors increases, the costs include the direct costs of increasing hardware as well as the accompanying installation, configuration, and maintenance costs. Therefore, the relationship between the number of redundant systems and the cost can be abstracted as a curve that increases rapidly and then slows down. The relationship between the cost and the number of sensors was obtained through actual investigation, and the cost function f(n) in the range of n∈[1, 10] (The reasons for the maximum value of n to 10 are mentioned in the next section) was obtained by using Taylor expansion for fitting:

(10) $$f(n)=f(a)+f^{\left(1\right)}(a)(n-a)+\frac{f^{\left(2\right)}(a)}{2!}{\left(n-a\right)}^{2}+\cdots +\frac{f^{\left(k\right)}(a)}{k!}{\left(n-a\right)}^{k}+R_{k}(n),$$

For the convenience of calculation, a is set to 0, and the first five terms of Taylor expansion are retained. After fitting, the f(n) is obtained:

(11) $$f(n)=0.00007n^{5}-0.0042n^{4}+0.0936n^{3}-0.9524n^{2}+4.6951n-2.4386,$$

**Figure 1:**
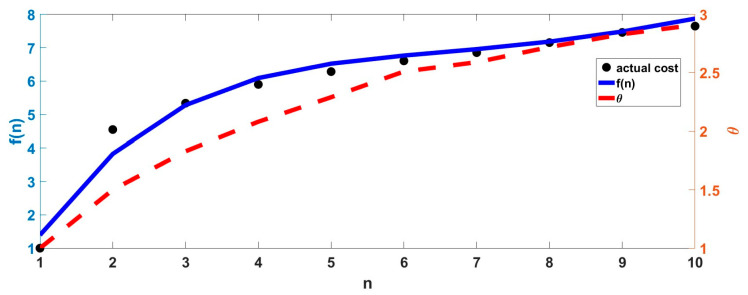
*f*(*n*) and *θ* change with the number of sensors.

**Figure 2:**
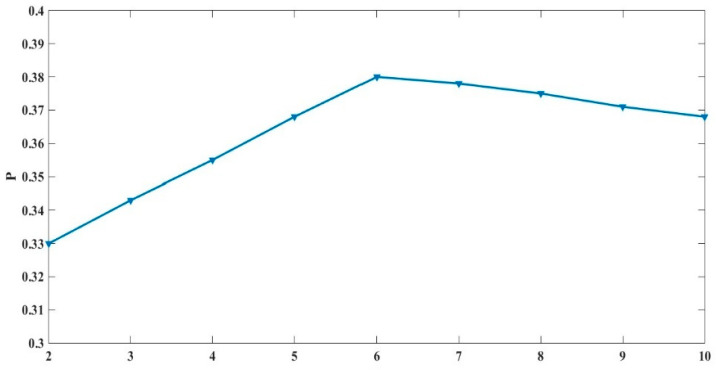
*P* changes with the number of sensors.

The relationship between cost function and reliability and the number of sensors is shown in Figure 1 below:

Therefore, the change in the performance constraint index P with the number of sensors n can be calculated, as shown in Figure 2 below:

The figure above shows that, when the number of sensors n is 6, the system performance constraint index P reaches the maximum, and then, the performance constraint index gradually decreases with the increase in the number of sensors. Therefore, the optimal number of sensors in the redundant configuration scheme is 6.

### 2.2. Redundancy Configuration Scheme for MEMS Sensors

**Figure 3:**
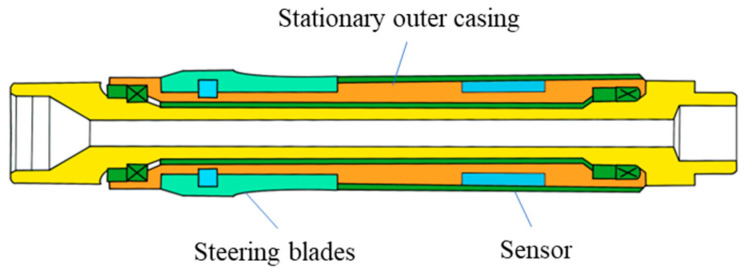
The structure of a static push-on rotary steerable tool.

Static push-on rotary steerable tools have found broad application in modern drilling and directional drilling practices, significantly enhancing drilling efficiency and enabling precise control over drilling trajectories. These tools primarily consist of a stationary outer casing and a rotating inner shaft (the dynamic component). During drilling operations, when a change in drilling direction is required, the control system activates steering blades, causing them to extend and press against the wellbore wall. At this point, the drilling assembly deflects under the push of the blades, while the stationary outer casing remains stationary, thereby providing a static environment for the installation of attitude measurement sensors. The structure of a static push-on rotary steerable tool is illustrated in Figure 3:

Based on the structure of the static push-type rotary steerable tool and the redundancy quantity of the MEMS sensor, a hollow hexagonal prism redundancy scheme is designed in this paper, which aims to ensure strength and stability while ensuring the flow of drilling fluid. This is because, to ensure continuous circulation of drilling fluid within the drill string, the redundant MEMS-IMU is designed as a hollow structure with multiple sensors mounted eccentrically. This eccentric mounting methodology encompasses two approaches: one involving cylindrical arrangement and the other based on a prismatic design. When the sensor is mounted around the cylinder, the installation of the sensor on the surface of the cylinder is more difficult, and because the relative installation position of the multi-sensor cannot be accurately determined on the cylinder, it will face a large installation error. Consequently, the prismatic configuration is chosen, which facilitates machining at fixed angles and, thereby, reduces both the complexity of installation and the resultant errors.

**Figure 4:**
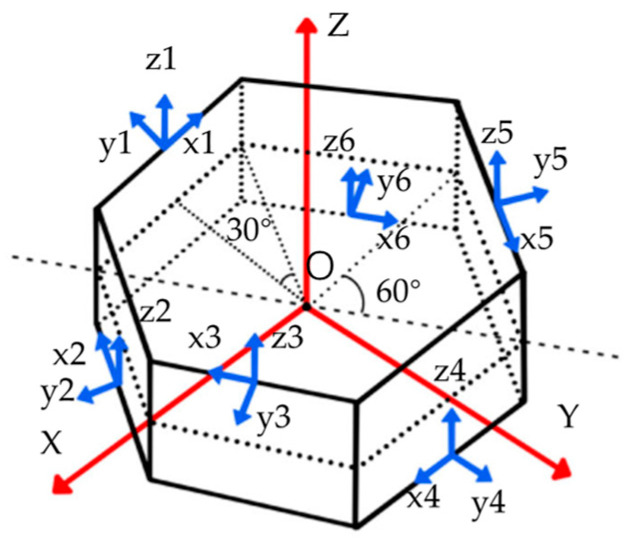
Hollow hexagonal prism redundancy configuration scheme.

From geometric relationships, it is known that the diameter of the inscribed circle of a regular polygon is directly proportional to the number of sides when the side length remains constant. As the number of sides increases, the radius of the inscribed circle gradually grows larger. The assumption that the side length of the polygon remains unchanged stems from the requirement that MEMS sensors must be mounted on the sides of the prism, and given that MEMS sensors have a certain dimensional footprint, the side length of the polygon must exceed the width of the MEMS sensors. There is an increasing demand for slim-hole drilling, typically defined as having a maximum diameter no greater than 150 mm, with some regions already defining slim-hole drilling as 98.4 mm [19]. As the number of sensors increases, the inscribed circle diameter corresponding to the redundant configuration scheme expands, and it is not suitable for slim-hole drilling. This highlights that the number of sensors cannot be increased indefinitely. Therefore, this study considers a maximum of 10 sensors. Because the planar configuration scheme is generally a polygon structure, although it can meet the requirements of internal hollow, the solution equation of sensor parameters may be underdetermined, resulting in an equation that cannot be solved. Therefore, this paper decides to design a spatial configuration scheme. The redundant configuration scheme design is shown in Figure 4:

The Cartesian coordinate system is established with the center of the prism as the origin O, the X-axis is perpendicular to the side of the prism, the Y-axis is perpendicular to the X-axis, and the Z and X and Y axes follow the right-hand rule upward. The MEMS sensors are numbered 1–6, and their centers are symmetrically installed at the midpoint of each edge. The axis of each sensor is shown in the figure above. The Z axis is consistent with the Z axis of the Cartesian coordinate system, the X and Y axes are at a certain angle with the X and Y axes of the Cartesian coordinate system, and the angle relationship is shown in the figure. Set $C_{x}$ as the X-axis installation matrix, representing the relative relationship between the X-axis orientation of each of the six sensors and the X-axis of the Cartesian coordinate system. Similarly, the installation matrix $C_{y}$ and $C_{z}$ can be obtained, which are expressed as follows:

(12) $$C_{x}=\left[\begin{array}{cccccc} -1 & 0 & 0 & 0 & 0 & 0\\ 0 & -\cos60^{\circ } & 0 & 0 & 0 & 0\\ 0 & 0 & \cos60^{\circ } & 0 & 0 & 0\\ 0 & 0 & 0 & 1 & 0 & 0\\ 0 & 0 & 0 & 0 & \cos60^{\circ } & 0\\ 0 & 0 & 0 & 0 & 0 & -\cos60^{\circ } \end{array}\right]\;C_{y}=\left[\begin{array}{cccccc} -1 & 0 & 0 & 0 & 0 & 0\\ 0 & -\sin30^{\circ } & 0 & 0 & 0 & 0\\ 0 & 0 & \sin30^{\circ } & 0 & 0 & 0\\ 0 & 0 & 0 & 1 & 0 & 0\\ 0 & 0 & 0 & 0 & \sin30^{\circ } & 0\\ 0 & 0 & 0 & 0 & 0 & -\sin30^{\circ } \end{array}\right]\;C_{z}=\left[\begin{array}{cccccc} 1 & 0 & 0 & 0 & 0 & 0\\ 0 & 1 & 0 & 0 & 0 & 0\\ 0 & 0 & 1 & 0 & 0 & 0\\ 0 & 0 & 0 & 1 & 0 & 0\\ 0 & 0 & 0 & 0 & 1 & 0\\ 0 & 0 & 0 & 0 & 0 & 1 \end{array}\right]$$

## 3. Data Fusion of Redundant MEMS-IMU

**Figure 5:**
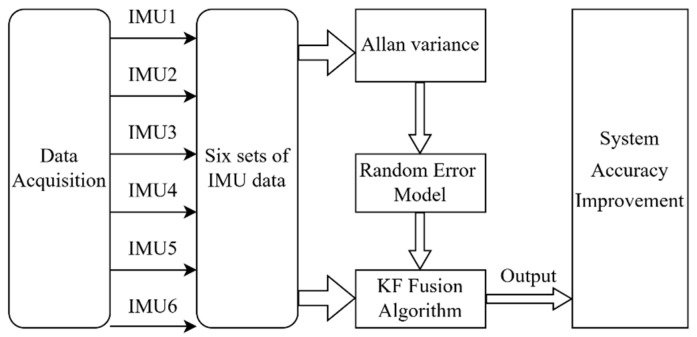
Redundant MEMS-IMU data fusion flowchart.

The specific process of multi-sensor data fusion is as follows: Firstly, the sensor data are analyzed by Allan variance to obtain noise parameters, and then, the sensor output error model is established. According to the model, a Kalman filter is used for data fusion. The system noise matrix and measurement noise matrix in the Kalman filter are set according to the noise parameters obtained by Allan variance [20,21,22,23], as shown in Figure 5 below:

The output error model of the MEMS gyroscope is shown as follows [24,25,26,27]:

(13) $$\left\{\begin{array}{c} y_{g}=\omega +b_{g}+n_{g}\\ \overset{\cdot }{b_{g}}=\varepsilon_{g} \end{array}\right.,$$

In the formula, $y_{g}$ is the gyroscope output, ω represents the true angular rate, $b_{g}$ represents a slow long-term drift process driven by the rate random walk $\varepsilon_{g}$, and $n_{g}$ represents the angular random walk.

The accelerometer output model is established by analogy with the error model of the gyroscope:

(14) $$\left\{\begin{array}{c} y_{a}=a+b_{a}+n_{a}\\ \overset{\cdot }{b_{a}}=\varepsilon_{a} \end{array}\right.,$$

where $y_{a}$ is the output of the accelerometer, a represents the real acceleration, $b_{a}$ represents a slow long-term drift process driven by the acceleration random walk $\varepsilon_{a}$, and $n_{a}$ represents the velocity random walk.

The Kalman data fusion filter is established according to the random error model of gyroscope output. Taking the X-axis as an example, the state equation and measurement equation of the Kalman filter are established as follows:

(15) $$\left\{\begin{array}{c} \overset{\cdot }{X_{g}(t)}=F_{g}X_{g}(t)+G_{g}W_{g}(t)\\ Z_{g}(t)=H_{g}X_{g}(t)+B_{g}V_{g}(t) \end{array}\right.,$$

In Formula (15), $X_{g}(t)$ stands for state vector, expressed as $X_{g}={\left[b_{g1},b_{g2},b_{g3},b_{g4},b_{g5},b_{g6},\omega \right]}^{T}$. The first six items of the state vector are the slow long-term drift of the six gyroscopes, and the seventh item is the X-axis angular rate of the Cartesian coordinate system established in the redundant system (as shown in Figure 4). Observed measurement is the projection of the X-axis output of each of the six gyros on the X-axis of the Cartesian coordinate system, obtained by Formula (13), expressed as $Z_{g}=C_{x}{\left[y_{g1},y_{g2},y_{g3},y_{g4},y_{g5},y_{g6}\right]}^{T}$. $C_{x}$ indicates the installation configuration matrix. $W_{g}(t)$ is the process noise, and $V_{g}(t)$ is the measurement noise; both are white noise. $W_{g}(t)={\left[\varepsilon_{g1},\varepsilon_{g2},\varepsilon_{g3},\varepsilon_{g4},\varepsilon_{g5},\varepsilon_{g6},0\right]}^{T}$, and $V_{g}(t)={\left[n_{g1},n_{g2},n_{g3},n_{g4},n_{g5},n_{g6}\right]}^{T}$. The six items in $V_{g}(t)$ represent the angular random walk of each of the six gyroscopes. The coefficient matrix of Kalman filtering is expressed as follows:

(16) $$F_{g}=\left[\begin{array}{ccccccc} 0 & 0 & 0 & 0 & 0 & 0 & 0\\ 0 & 0 & 0 & 0 & 0 & 0 & 0\\ 0 & 0 & 0 & 0 & 0 & 0 & 0\\ 0 & 0 & 0 & 0 & 0 & 0 & 0\\ 0 & 0 & 0 & 0 & 0 & 0 & 0\\ 0 & 0 & 0 & 0 & 0 & 0 & 0\\ 0 & 0 & 0 & 0 & 0 & 0 & 0 \end{array}\right]\;G_{g}=\left[\begin{array}{ccccccc} 1 & 0 & 0 & 0 & 0 & 0 & 0\\ 0 & 1 & 0 & 0 & 0 & 0 & 0\\ 0 & 0 & 1 & 0 & 0 & 0 & 0\\ 0 & 0 & 0 & 1 & 0 & 0 & 0\\ 0 & 0 & 0 & 0 & 1 & 0 & 0\\ 0 & 0 & 0 & 0 & 0 & 1 & 0\\ 0 & 0 & 0 & 0 & 0 & 0 & 1 \end{array}\right]\;H_{g}=\left[\begin{array}{ccccccc} 1 & 0 & 0 & 0 & 0 & 0 & 1\\ 0 & 1 & 0 & 0 & 0 & 0 & 1\\ 0 & 0 & 1 & 0 & 0 & 0 & 1\\ 0 & 0 & 0 & 1 & 0 & 0 & 1\\ 0 & 0 & 0 & 0 & 1 & 0 & 1\\ 0 & 0 & 0 & 0 & 0 & 1 & 1 \end{array}\right]\;B_{g}=\left[\begin{array}{cccccc} 1 & 0 & 0 & 0 & 0 & 0\\ 0 & 1 & 0 & 0 & 0 & 0\\ 0 & 0 & 1 & 0 & 0 & 0\\ 0 & 0 & 0 & 1 & 0 & 0\\ 0 & 0 & 0 & 0 & 1 & 0\\ 0 & 0 & 0 & 0 & 0 & 1 \end{array}\right]$$

After discretization, Formula (15) can be expressed as follows:

(17) $$\left\{\begin{array}{c} \Phi_{k/k-1}=I_{6+1}\\ \Gamma_{k-1}=tI_{6+1}\\ W_{k}={\left[\varepsilon_{g1}\left(k\right),\varepsilon_{g2}\left(k\right),\varepsilon_{g3}\left(k\right),\varepsilon_{g4}\left(k\right),\varepsilon_{g5}\left(k\right),\varepsilon_{g6}\left(k\right),0\right]}^{T}\\ V_{k}={\left[n_{g1}\left(k\right),n_{g2}\left(k\right),n_{g3}\left(k\right),n_{g4}\left(k\right),n_{g5}\left(k\right),n_{g6}\left(k\right)\right]}^{T} \end{array}\right.$$

where *t* is the sampling period, $W_{k}$ is the system drive white noise sequence, and $V_{k}$ is the measurement noise sequence.

(18) $$\left\{\begin{array}{c} E[W_{k}]=0,Cov[W_{k},W_{j}]=E[W_{k}W_{j}{\;\;}^{T}]=Q\delta_{kj}\\ E[V_{k}]=0,Cov[V_{k},V_{j}]=E[V_{k}V_{j}{\;\;}^{T}]=R\delta_{kj}\\ Cov[W_{k},V_{j}]=E[W_{k}V_{j}{\;\;}^{T}]=0 \end{array}\right.$$

Among them,

(19) $$Q=\left[\begin{array}{cc} Q_{\varepsilon } & 0\\ 0 & 0 \end{array}\right],R=[Q_{n}]$$

*Q* and *R* are the covariance matrices of system noise and measurement noise, respectively. $Q_{\varepsilon }$ is the covariance matrix of the rate random walk noise vector, and $Q_{n}$ is the covariance matrix of the angular random walk noise vector. In this paper, the six MEMS-IMUs are packaged independently; therefore, it is assumed that there is no correlation between the random errors of the six sensors, so there are the following:

(20) $$Q_{\varepsilon }={\left[\begin{array}{cccc} \sigma_{\varepsilon 1}^{2} & 0 & \cdots & 0\\ 0 & \sigma_{\varepsilon 2}^{2} & \cdots & 0\\ \vdots & \vdots & \ddots & \vdots \\ 0 & 0 & \cdots & \sigma_{\varepsilon 6}^{2} \end{array}\right]}_{6\times 6}R={\left[\begin{array}{cccc} \sigma_{n1}^{2} & 0 & \cdots & 0\\ 0 & \sigma_{n2}^{2} & \cdots & 0\\ \vdots & \vdots & \ddots & \vdots \\ 0 & 0 & \cdots & \sigma_{n6}^{2} \end{array}\right]}_{6\times 6}$$

where $\sigma_{\varepsilon i}^{2}$ represents the rate random walk noise variance of gyroscope i, and $\sigma_{ni}^{2}$ represents the angular random walk variance of gyroscope i.

The acceleration fusion filter is established by analogy with the angular velocity fusion filter. The Kalman data fusion filter is established according to the random error model of the accelerometer output. Also, taking the X-axis as an example, the state equation and measurement equation of the Kalman filter are established as follows:

(21) $$\left\{\begin{array}{c} \overset{\cdot }{X_{a}(t)}=F_{a}X_{a}(t)+G_{a}W_{a}(t)\\ Z_{a}(t)=H_{a}X_{a}(t)+B_{a}V_{a}(t) \end{array}\right.,$$

State vector $X_{a}={\left[b_{a1},b_{a2},b_{a3},b_{a4},b_{a5},b_{a6},a\right]}^{T}$, and a is the X axis acceleration of the Cartesian coordinate system established in the redundant system. Observed measurement is the projection of the X-axis output of each of the six accelerometers on the X-axis of the Cartesian coordinate system, expressed as $Z_{a}=C_{x}{\left[y_{a1},y_{a2},y_{a3},y_{a4},y_{a5},y_{a6}\right]}^{T}$. $W_{a}(t)$ is the process noise, and $V_{a}(t)$ is the measurement noise. $W_{a}(t)={\left[\varepsilon_{a1},\varepsilon_{a2},\varepsilon_{a3},\varepsilon_{a4},\varepsilon_{a5},\varepsilon_{a6},0\right]}^{T}$, and $V_{a}(t)={\left[n_{a1},n_{a2},n_{a3},n_{a4},n_{a5},n_{a6}\right]}^{T}$. The covariance matrix of system noise and measurement noise is and, respectively, expressed as follows:

(22) $$Q_{a}=\left[\begin{array}{cc} Q_{\varepsilon a} & 0\\ 0 & 0 \end{array}\right],R_{a}=[Q_{na}]$$

$Q_{\varepsilon }$ is the covariance matrix of the acceleration random walk noise vector, and $Q_{n}$ is the covariance matrix of the velocity random walk noise vector.

## 4. Experimental Analysis of Redundant MEMS-IMU System

**Figure 6:**
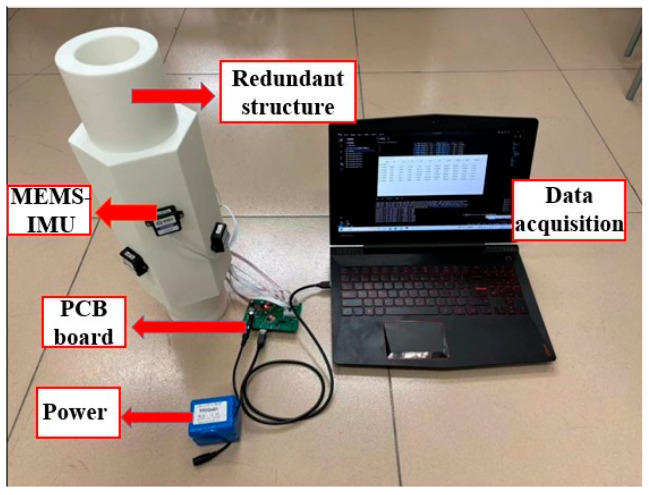
Overall picture of the experimental device.

The MEMS-IMU is connected to the PCB board through the RS-232 serial cable for data transmission, and data are read and saved at the PC end. The overall experimental device is shown in Figure 6, and the selected sensor parameters are shown in Table 1.

**Figure 7:**
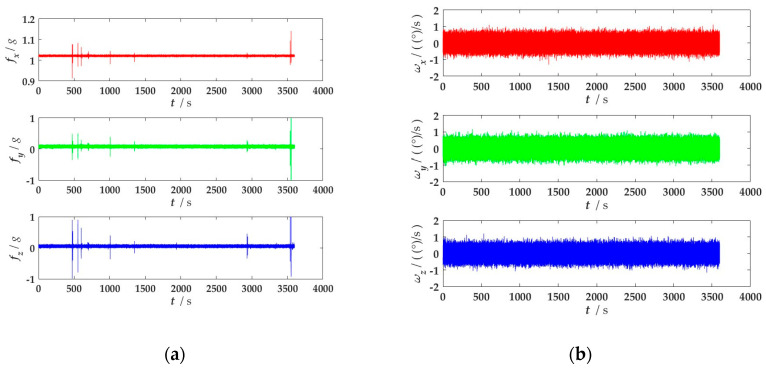
Raw data of a single IMU: (**a**) Three-axis accelerometer output; (**b**) Three-axis gyroscope output.

**Figure 8:**
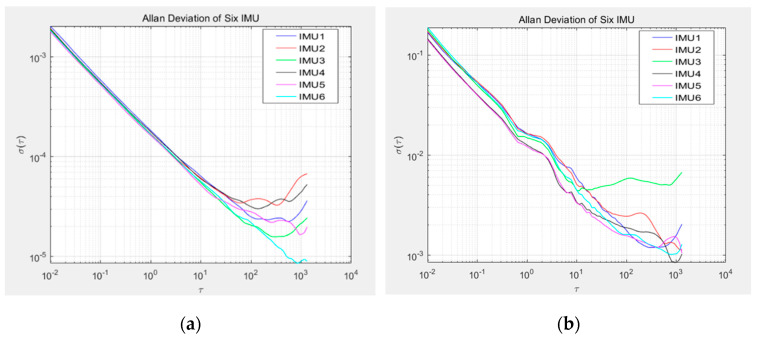
X-axis Allan variance analysis chart for six MEMS-IMUs: (**a**) accelerometer; (**b**) gyroscope.

First, data acquisition from the MEMS-IMU is performed at room temperature. In accordance with the environmental requirements of the device in use, a preheating process for the MEMS-IMU is carried out, which involves powering on the device and allowing the sensors to warm up for 20 to 30 min, ensuring that their outputs reach a stable state. During this warm-up period, the data outputted by the sensors are not collected. After the preheating, the sampling frequency of each MEMS-IMU is set to 100 Hz, and the sampling time is 1 h. A total of approximately 6 × 360,000 sets of sample data are obtained. The original data output is shown in Figure 7 (taking an IMU as an example):

The Allan variance method was used to analyze the collected data, and the Allan deviation log-log curve was obtained, as shown in Figure 8 (this paper only takes the X-axis data of each IMU as an example):

The Allan standard deviation logarithmic curve was fitted, and the fitting coefficients of each major error were calculated, as shown in Table 2 and Table 3:

According to the error coefficient table of the MEMS-IMU above, the parameters of the gyroscope filter can be calculated by Formula (20) as follows:

(23) $$Q_{\varepsilon }=\left[\begin{array}{cccccc} 37.59 & 0 & 0 & 0 & 0 & 0\\ 0 & 15.00 & 0 & 0 & 0 & 0\\ 0 & 0 & 14.42 & 0 & 0 & 0\\ 0 & 0 & 0 & 12.78 & 0 & 0\\ 0 & 0 & 0 & 0 & 4.38 & 0\\ 0 & 0 & 0 & 0 & 0 & 14.09 \end{array}\right]\times \deg/h^{\frac{3}{2}}\;R=\left[\begin{array}{cccccc} 0.9427 & 0 & 0 & 0 & 0 & 0\\ 0 & 1.0752 & 0 & 0 & 0 & 0\\ 0 & 0 & 0.9060 & 0 & 0 & 0\\ 0 & 0 & 0 & 0.7778 & 0 & 0\\ 0 & 0 & 0 & 0 & 0.7529 & 0\\ 0 & 0 & 0 & 0 & 0 & 0.8536 \end{array}\right]\times \deg/\sqrt{h}$$

The parameters of the accelerometer filter are as follows:

(24) $$Q_{\varepsilon a}=\left[\begin{array}{cccccc} 0.3349 & 0 & 0 & 0 & 0 & 0\\ 0 & 0.7550 & 0 & 0 & 0 & 0\\ 0 & 0 & 0.2573 & 0 & 0 & 0\\ 0 & 0 & 0 & 0.5254 & 0 & 0\\ 0 & 0 & 0 & 0 & 0.1952 & 0\\ 0 & 0 & 0 & 0 & 0 & 0.1072 \end{array}\right]\times m/s/h^{\frac{3}{2}}\;R_{a}=\left[\begin{array}{cccccc} 0.0110 & 0 & 0 & 0 & 0 & 0\\ 0 & 0.0102 & 0 & 0 & 0 & 0\\ 0 & 0 & 0.0100 & 0 & 0 & 0\\ 0 & 0 & 0 & 0.0111 & 0 & 0\\ 0 & 0 & 0 & 0 & 0.0100 & 0\\ 0 & 0 & 0 & 0 & 0 & 0.0104 \end{array}\right]\times m/s/\sqrt{h}$$

**Figure 9:**
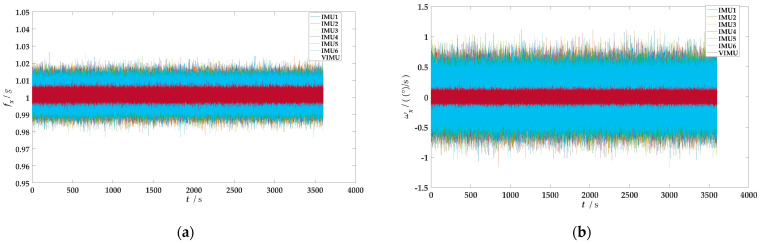
Comparison between the output of the VIMU and each IMU X-axis: (**a**) accelerometer; (**b**) gyroscope.

**Figure 10:**
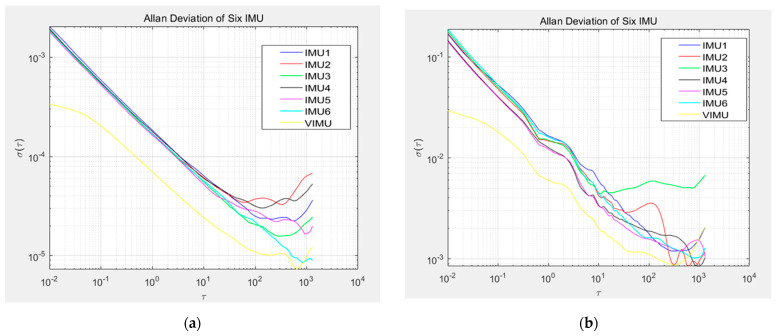
X-axis Allan variance comparison between VIMU and six IMUs raw data: (**a**) accelerometer; (**b**) gyroscope.

A set of data output after six groups of IMU data fusion is obtained by using the designed filter, which is recorded as VIMU(Virtual-IMU). The comparison with the six groups of original data is shown in Figure 9 (taking the accelerometer and gyroscope X-axis output as an example):

As can be observed in the above figure, the drift phenomenon of the output data of the virtual IMU is significantly reduced compared with the original data of the six IMUs, and the interference noise is also significantly reduced. Allan variance analysis is carried out on the output data of the virtual IMU, and the difference map is fitted to obtain the error coefficient. In this paper, velocity/angular random walk and bias instability are taken as comparison indicators before and after data fusion. The Allan variance graph of the virtual IMU output is compared with the variance of the original data, as shown in Figure 10 (taking the X-axis data as an example):

Comparison of the mean error coefficients of each axis between the VIMU and single IMU is shown in Table 4 and Table 5:

According to the Allan variance comparison chart, the drift phenomenon of the virtual IMU output will be greatly improved compared with that of the single IMU output, and the quantitative conclusion can be obtained: The velocity random walk of the virtual accelerometer decreases by 54.07% in the X-axis direction, 62.56% in the Y-axis direction, and 59.54% in the Z-axis direction. The bias instability of the six accelerometers decreases by 55.27% in the X-axis direction, 49.04% in the Y-axis direction, and 57.95% in the Z-axis direction. Compared with the average gyroscope random walk in the six IMUs, the virtual gyroscope angular random walk decreases by 55.00% in the X-axis, 60.47% in the Y-axis, and 59.54% in the Z-axis. The bias instability is reduced by 42.71% in the X-axis direction, 40.66% in the Y-axis direction, and 29.88% in the Z-axis direction compared to the average zero deviation of the six gyroscopes. The effectiveness of the virtual IMU technique and Kalman filter design in this paper is proved.

## 5. Discussion

Although the research in this paper has achieved phased results in improving the application accuracy and reliability of MEMS sensors in the field of geological drilling, there are still some limitations and room for improvement. Future work can be further studied from the following aspects:

1. The data fusion algorithm proposed in this paper is only applicable to static conditions, but in dynamic cases, it faces the problem of different sensor sensitive axes. How to perform data fusion under different sensor sensitive axes is the next research focus.
2. This paper only studies and analyzes the random error of MEMS sensors as an indicator of accuracy improvement, and the installation error of a multi-sensor redundancy system has a huge impact on accuracy. Subsequently, the deterministic error of this sensor redundancy system will be taken as the research object, and a deterministic error model will be established to compensate the installation error.
3. The cost function derived in this paper is not universal but provides a way to evaluate the optimal number of redundancies. The cost function should be analyzed on a case-by-case basis, and its specific shape depends on the technical details of the different fields, economies of scale, and the specific requirements of the system design.

## 6. Conclusions

During drilling operations underground, the reliability and measurement accuracy of a single MEMS-IMU can be difficult to ensure. This paper innovatively proposes a redundancy method for MEMS-IMUs tailored to the special structure of downhole drilling tools. By examining the relationship between the reliability of sensor redundancy systems and their cost, an optimal number of six sensors for the redundancy system is determined. Considering the actual working conditions of downhole drilling tools, a hollow hexagonal prism-shaped redundant configuration scheme is designed, which ensures the circulation of drilling fluid within the drill string.

Furthermore, by employing data fusion algorithms, data from multiple low-precision sensors are combined, thereby enhancing the measurement precision of the sensors. This redundancy configuration scheme, while considering both cost and size constraints, enhances the reliability and accuracy of the downhole attitude measurement unit, meeting the stringent requirements for precision and reliability in underground operations. Ultimately, it ensures that the measurement needs in challenging subterranean environments are effectively addressed, advancing the capabilities of drilling technology.

## Figures and Tables

**Table 1:** Performance specifications of the sensor.

Accelerometer	Minimum Value	Typical Value	Maximum Value
Range (g)	−6	/	+6
Bias instability (μg)	5	10	15
Velocity random walk (m/s/√h)	0.03	0.05	0.1
Bandwidth (Hz)	/	50	/
**Gyroscope**	**Minimum Value**	**Typical Value**	**Maximum Value**
Range (°/s)	−400	/	+400
Bias instability (°/h)	3	4	5
Angular random walk (°/h)	0.2	0.3	0.5
Bandwidth (Hz)	/	50	/

**Table 2:** X-axis error coefficient of MEMS-IMU accelerometer.

Error Term	N (m/s/√h)	B (μg)	K (m/s/h^3/2^)
accelerometer 1	0.0110	35.948	0.3349
accelerometer 2	0.0102	57.416	0.7550
accelerometer 3	0.0100	23.768	0.2573
accelerometer 4	0.0111	45.400	0.5254
accelerometer 5	0.0100	34.653	0.1952
accelerometer 6	0.0104	14.484	0.1072

**Table 3:** X-axis error coefficient of MEMS-IMU gyroscope.

Error Term	N (°/√h)	B (°/h)	K (°/h^3/2^)
gyroscope 1	0.0110	35.948	0.3349
gyroscope 2	0.0102	57.416	0.7550
gyroscope 3	0.0100	23.768	0.2573
gyroscope 4	0.0111	45.400	0.5254
gyroscope 5	0.0100	34.653	0.1952
gyroscope 6	0.0104	14.484	0.1072

**Table 4:** Comparison between the errors of the VIMU and the mean of the six accelerometers.

Errors	N (m/s/√h)			B (μg)		
	x	y	z	x	y	z
Mean of six IMUs	0.0104	0.0111	0.0142	35.278	46.212	43.135
VIMU	0.0041	0.0041	0.0044	15.779	23.550	18.140
Error reduction	54.1%	62.6%	69.1%	55.3%	49.0%	57.9%

**Table 5:** Comparison between the errors of the VIMU and the mean of the six gyroscopes.

Errors	N (°/√h)			B (°/h)		
	x	y	z	x	y	z
Mean of six IMUs	0.8847	0.6223	0.7313	9.0340	4.3135	6.2499
VIMU	0.3981	0.2460	0.2959	6.3210	2.5595	4.3823
Error reduction	55.0%	60.5%	59.5%	42.7%	40.7%	29.9%

## Data Availability

The original contributions presented in this study are included in the article.
